# Mitochondrial DNA copy number in patients with systemic sclerosis

**DOI:** 10.3389/fmolb.2023.1313426

**Published:** 2023-12-14

**Authors:** Anastasia I. Bogatyreva, Elena V. Gerasimova, Tatiana V. Kirichenko, Yuliya V. Markina, Taisiya V. Tolstik, Diana G. Kiseleva, Tatiana V. Popkova, Alexander M. Markin

**Affiliations:** ^1^ Laboratory of Cellular and Molecular Pathology of Cardiovascular System, Avtsyn Research Institute of Human Morphology of FSBSI “Petrovsky National Research Centre of Surgery”, Moscow, Russia; ^2^ Department of Systemic Rheumatic Diseases, VA Nasonova Research Institute of Rheumatology, Moscow, Russia; ^3^ Faculty of Biology, Department of Biophysics, Lomonosov Moscow State University, Moscow, Russia; ^4^ Medical Institute, Peoples’ Friendship University of Russia named after Patrice Lumumba (RUDN University), Moscow, Russia

**Keywords:** DNA, mitochondrial, monocytes, systemic sclerosis, autoimmunity, inflammation

## Abstract

**Introduction:** Systemic scleroderma (SSc) is a chronic autoimmune disease of inflammatory origin. Mitochondrial dysfunction is considered as an important mechanism in the pathogenesis of SSc. Currently mitochondrial DNA (mtDNA) copy number is used as a surrogate marker of mitochondrial dysfunction. Previous studies demonstrate that innate immune cells are important participants in inflammatory and fibrotic processes in SSc. The aim of the study was to evaluate the number of mtDNA copies in CD14^+^ monocytes and whole blood of patients with SSc in comparison with healthy individuals.

**Methods:** Absolute mtDNA copy number was measured using digital PCR. It was found that the number of mtDNA copies in CD14^+^ monocytes was significantly higher in patients with SSc compared to control, while the number of mtDNA copies in the whole blood did not have significant differences.

**Results:** The correlation analysis revealed an inverse association of mtDNA copy number with disease duration and the relationship between pro-inflammatory activation of CD14^+^ monocytes in terms of LPS-stimulated IL-6 secretion and mtDNA copy number. At the same time, basal and LPS-stimulated secretion of IL-6 by cultured CD+ monocytes were significantly higher in SSc group in comparison with control.

**Discussion:** The study results suggest that increase of mtDNA copy number in CD14^+^ monocytes is a possible mechanism to maintain the reduced function of defective mitochondria in monocytes from patients with SSc associated with the development and progression of SSc.

## 1 Introduction

Systemic sclerosis (SSc) is an autoimmune disease characterized by fibrosis of the skin and internal organs, as well as vasculopathy. Despite of the fact that SSc is not a widespread disease, this pathology has the highest mortality rate among all rheumatic diseases (Volkmann et al., 2023). The etiology of SSc remains unknown; probably, the combined effect of genetic predisposition and external factors plays a decisive role in the induction of the disease (Rosendahl et al., 2022).

Mitochondria are considered as an important link in the regulation of both innate and adaptive immunity; mitochondrial dysfunction leads to disruption of the immune system (Xu et al., 2020). As a result of mitochondrial dysfunction, there is a disruption in the electron transport chain leading to an increase in reactive oxygen species, that, in turn, affects the secretion of pro-inflammatory cytokines, which leads to the activation and migration of immune cells to sites of inflammation (Zank et al., 2018). Mitochondrial DNA (mtDNA) copy number is considered as a surrogate marker of mitochondrial dysfunction. Changes in the amount of mtDNA can lead to increased oxidative stress and contribute to the development of inflammation (Malik and Czajka, 2013). A number of studies have shown that the mtDNA copy number in patients with autoimmune rheumatic diseases (ARDs) differs from healthy individuals (Svendsen et al., 2019; Giaglis et al., 2021).

Monocytes play an important role in the pathogenesis of SSc. Most peripheral blood CD14^+^ monocytes differentiate into tissue macrophages, which secrete proinflammatory cytokines and chemokines, leading to chronic inflammation in patients with SSc (Mathai et al., 2010). It is known that the number of CD14^+^ monocytes is increased in patients with SSc (Higashi-Kuwata et al., 2010). Increased level of circulating pro-inflammatory cytokines in SSc was demonstrated in several clinical trials (Lescoat et al., 2021). In particular, high serum levels of pro-inflammatory cytokine interleukin-6 (IL-6) and important role of IL-6 in the pathogenesis of SSc were demonstrated, that determined the use of IL-6 as a target for biological therapy of SSc (Sambo et al., 1999). Mitochondrial dysfunction in circulating monocytes may cause dysfunction of monocytes and lead to the progression of chronic inflammation in SSc.

The purpose of this study was to investigate the copy number of mtDNA in CD14^+^ monocytes and all cell populations circulating in the blood of patients with SSc and apparently healthy individuals and to assess the association of mtDNA copy number with pro-inflammatory status of monocytes assessed by IL-6 secretion by cultured monocytes under basal and pro-inflammatory conditions, mitochondrial activity, and clinical characteristics of patients with SSc.

## 2 Materials and methods

### 2.1 Study design

The study included 25 patients with SSc and a control group of 25 apparently healthy individuals without SSc and other autoimmune or chronic inflammatory diseases. SSc patients included in the study did not receive glucocorticoid therapy. Exclusion criteria were age younger than 20 years or older than 70 years; the presence of diabetes mellitus, cancer, decompensated renal or liver failure, chronic cardiovascular failure NYHA class III-IV.

Table 1 demonstrates the general characteristics, clinical and laboratory manifestations of SSc patients. Most patients (80%) were diagnosed with a limited form of SSc. The disease activity score for SSc patients was adapted by the European Scleroderma Study Group and the SSc Clinical Trials Consortium (Valentini et al., 2001). Deterioration of the skin (Δ skin), Raynaud’s syndrome (Δ vascular) and cardiovascular manifestations (Δ heart/lungs) in the month preceding hospitalization were identified when interviewing the patient. The presence of scleredema, i.e., swelling of the skin of the hands, digital necrosis and arthritis, was determined during examination. The study of external respiration function was carried out using a Master Screen PFT device (Viasys Healthcare, Germany). Spirometry and body plethysmography were performed to determine standard static and dynamic ventilation indicators. The diffusion capacity of the lungs was assessed by the single breath method and expressed as a percentage of the expected value; 80%–120% was considered normal values. The level of complement components (C3 and/or C4) was determined in the patient’s blood serum by immunonephelometry (BN ProSpec, Siemens); hypocomplementemia was considered as a decrease in the level below normal for each indicator; normal values were considered for C3 0.9–1.8 g/L, for C4 0.1–0.4 g/L. The clinical and laboratory parameters that made up the SSc activity index were recorded and scored, after which the overall SSc activity index was calculated. The mean value of the activity index was 2.1 (1.8) points. A low activity (<3 points) was detected in 76% of SSc patients, a high activity (≥3 points) was detected in 24% of patients (24%).

**TABLE 1:** General characteristics, clinical and laboratory manifestations of SSc patients.

Characteristics		Value
Age, years		50 (12)
Gender, f/m, n (%)		22 (88)/3 (12)
Disease duration, years		6.2 (6.6)
Types of SSc, n (%)	Limited	20 (80)
	Diffuse	5 (20)
Activity index, points		2.1 (1.8)
Skin thickening of the fingers of both hands extending proximal to the metacarpophalangeal joints, n (%)		20 (80)
Skin thickening of the fingers, n (%)	Puffy fingers	12 (48)
	Sclerodactyly of the fingers (distal to metacarpophalangeal joints but proximal to the proximal interphalangeal joints)	8 (32)
Fingertip lesions, n (%)	Digital tip ulcers	9 (36)
	Fingertip pitting scars	12 (48)
Telangiectasia, n (%)		11 (44)
Abnormal nailfold capillaries, n (%)		21 (84)
Pulmonary arterial hypertension, n (%)		3 (12)
Interstitial lung disease, n (%)		5 (20)
Raynaud’s phenomenon, n (%)		20 (80)
SSc related autoantibodies, n (%)	Anti-topoisomerase I	8 (32)
	Anticentromere	12 (48)
	Anti-RNA polymerase III	2 (8)

### 2.2 CD14^+^ monocytes isolation

CD14^+^ monocytes were isolated from whole blood samples using a standard method for isolating the leukocyte fraction in a Ficoll gradient, followed by the selection of CD14^+^ cells by magnetic separation on columns (Miltenyi Biotec, United States) using paramagnetic nanoparticles (Miltenyi Biotec, United States).

### 2.3 DNA isolation

Isolation of DNA from CD14^+^ monocytes and whole blood was carried out using the ExtractDNA Blood and Cells kit (Evrogen, Russia) according to the manufacturer’s protocol. The concentration and purity of the obtained DNA were determined using a BIOSPEC-NANO spectrophotometer (Shimadzu, Japan).

### 2.4 Quantitative determination of mtDNA copies

The copy number of mtDNA and nDNA was determined by digital PCR on a QIAcuity Eight instrument (Qiagen, Germany). For amplification, primers (Synthol, Russia) and the reaction mixture QuantiFast SYBR Green Master Mix (Qiagen, Germany) were used. PCR was performed for 42 cycles, with pre-denaturation for 2 min at 95°C for the first cycle, the next 40 cycles included denaturation for 15 s at 95°C, annealing for 30 s at 60°C and elongation for 30 s at 72°C, the final cycle (temperature extension) lasted 5 min at 40°C. For each test sample, the PCR reaction was performed at least three times.

PCR reactions were performed in a total volume of 12 μL using 5 μL of genomic DNA, 0.4 μL of forward and reverse primers, 4 μL of QuantiFast SYBR Green Master Mix and 2.2 μL of deionized water.

To determine the number of mtDNA and nDNA copies, primers F MT-ND4, R MT-ND4, F NCOA3, R NCOA3 were used. Nucleotide sequence F MT-ND4: 5′-cca​ttc​tcc​tcc​tat​ccc​tca​ac-3′, R MT-ND4: 5′-cac​aat​ctg​atg​ttt​tgg​tta​aac​tat​att​t-3′, F NCOA3: 5′-gag​ttt​cct​gga​caa​atg​ag-3′, R NCOA3: 5′-cat​tgt​ttc​ata​tct​ctg​gcg-3′. Primers were selected based on literature data (Bai and Wong, 2005; Gu et al., 2013). The value of mtDNA copy number per cell that was used for analysis was calculated as a ratio of mtDNA and nDNA copies (Shoop et al., 2022).

The researchers were blinded to the group status and clinical characteristics of the study participants.

### 2.5 Analysis of IL-6 secretion by cultured CD14^+^ monocytes

CD14^+^ monocytes obtained by immunomagnetic separation as described above, were seeded into two wells of a 48-well plate at a rate of 500,000 cells per well and cultured in 0.5 mL of serum-free X-VIVO medium (Lonza, Germany) containing L-glutamine, gentamicin and phenol red at 37°C. In the first well cells were cultivated under basal conditions. In the second well, the inflammatory response of monocytes was stimulated by the addition of LPS from *Escherichia coli*, serotype 026:B6 (Sigma-Aldrich, Israel) at a concentration 1 μg/mL. The cells were cultured for 24 h, then the samples of culture fluid were collected and stored at −70°C for analysis of IL-6 secretion. The concentration of IL-6 in culture fluid samples was determined by ELISA using a commercial Human IL-6 DuoSet ELISA kit (R&D Systems, United States).

### 2.6 Analysis of mitochondrial activity

The MitoTracker Orange staining assay was performed to evaluate the mitochondrial activity. The isolated CD14^+^ monocytes in the amount of 1 million cells were resuspended in the medium in eppendorf-type test tubes with the addition of MitoTracker orange dye (Thermo Fisher Scientific, United States) at a concentration of 2 μL/mL and were incubated at 37°C with an open lid for 30 min. Then the cells were centrifugated at 8,000–13,000 g for 6 min. The fluorescence intensity was analyzed with flow cytometer MACSQuant VYB (Miltenyi Biotec, Germany). The obtained mean values of fluorescence intensity were used for statistical processing.

### 2.7 Statistical analysis

The sample size was estimated using Cochran’s equation together with a correction for small populations (2–12 cases per million people) of a known size, to calculate sample size for Precision Level 0.05, Confidence Level 95%, Estimated Proportion 0.5. Statistical analysis of the obtained data was carried out using the R programming language for statistical computing. The Mann-Whitney *U* test was used to assess a statistically significant difference between the two samples. The data on clinical characteristics of study participants are presented as mean value and standard deviation [Mean (SD)]. The obtained values of mtDNA copy number are presented as median and quartiles [Me (Q1, Q3)]. Pearson correlation analysis was performed to investigate the association of mtDNA copy number with clinical characteristics of SSc study participants.

## 3 Results

A total of 50 participants were included in the study: 25 patients with SSc and 25 control participants without SSc. The mean age of study participants was 47.9 (12.1) years old in the group of SSc patients and 49.7 (9.7) years old in the control group, *p* = 0.556. Study groups were also matched by gender, 80% of SSc patients and 72% of control study participants were female, *p* = 0.518. The study was conducted in accordance with the Declaration of Helsinki of 1975 and its revised version of 2013. The study protocol was approved by the Local Ethics Committee of the Nasonova Research Institute of Rheumatology at 10 February 2022. All participants provided written informed consent to participate in the study.

**FIGURE 1:**
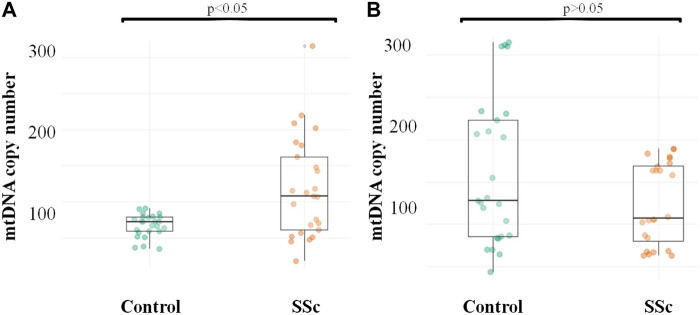
mtDNA copy number in CD14^+^ monocytes and whole blood of patients with SSc and healthy study participants **(A)** Quantification of CD14^+^ monocyte mtDNA; **(B)** Quantification of mtDNA of all cell populations circulating in the blood.

The number of mtDNA copies was measured in CD14^+^ monocytes and all cell populations circulating in the blood of patients with SSc and apparently healthy individuals. It was shown that the absolute number of mtDNA copies of CD14^+^ monocytes was significantly higher in the group of patients with SSc, 108 [60–162] compared to the control group, 72 [59–79], *p* = 0.011*, while the absolute number of mtDNA copies of all cell populations circulating in the blood was decreased in SSc patients, 109 [72–171] than in control individuals, 128 [85–227], but the difference between groups was not significant, *p* = 0.171. The obtained data are presented in Figure 1.

**FIGURE 2:**
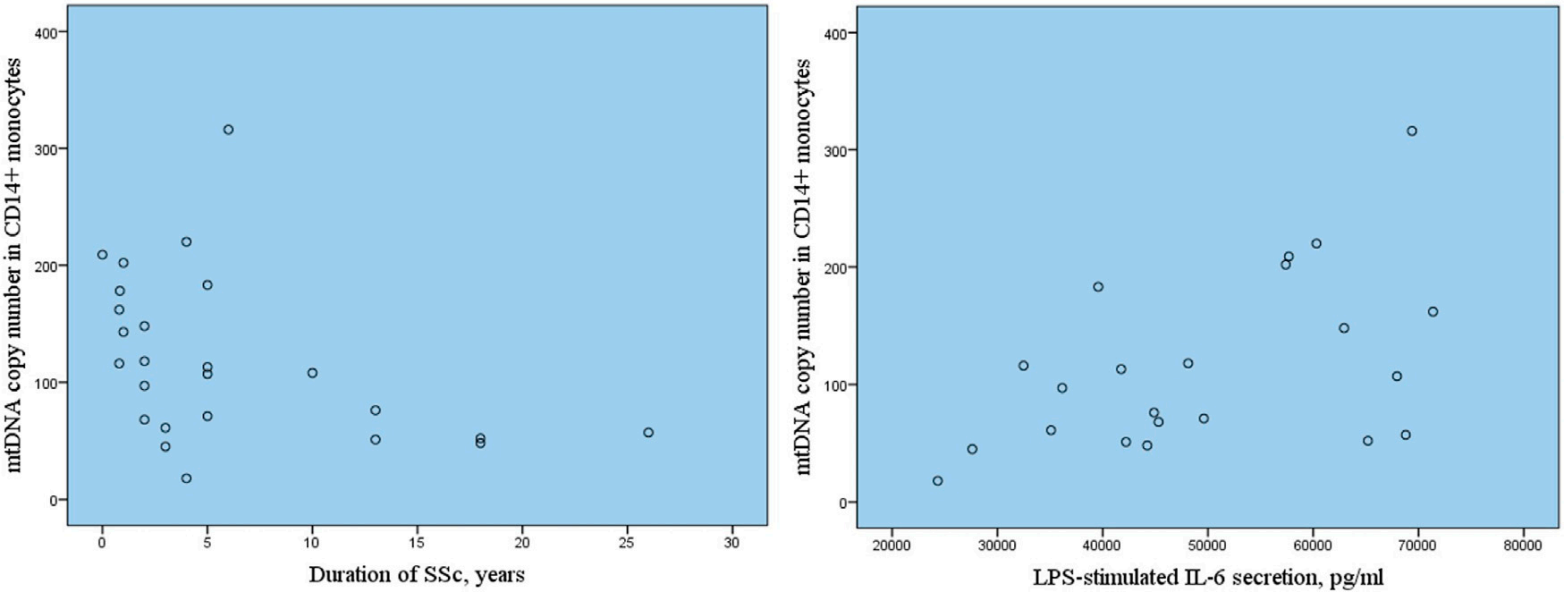
Correlation of mtDNA copy number in CD14^+^ monocytes with SSc duration and LPS-stimulated secretion of pro-inflammatiry cytokine IL-6.

The analysis of association of mtDNA copy number with clinical characteristics of patients with SSc revealed the significant inverse correlation of mtDNA copy number in CD14^+^ monocytes with disease duration with *r* = −0.420, *p* = 0.037* (Figure 2). The inverse relationship was also observed between C14+ mtDNA copy number and disease activity, however, the correlation was not statistically significant, *r* = −0.315, *p* = 0.254. No association of mtDNA copy number in CD14^+^ monocytes and other clinical characteristics of patients with SSc was observed as well as no clinical characteristics correlated with mtDNA copy number in whole blood samples of patients with SSc.

Next the association of mtDNA copy number in CD14^+^ monocytes with inflammatory activation of monocytes assessed by basal and LPS-stimulated secretion of pro-inflammatory cytokine Il-6 was investigated. Table 2 presents the results of measurements of Il-6 secretion by cultured monocytes of study participants with SSc in comparison with apparently healthy individuals. It was demonstrated that basal and LPS-stimulated secretion of IL-6 was significantly higher in SSc group in comparison with control group. The correlation analysis demonstrated that increased LPS-stimulated secretion of IL-6 by cultured CD14^+^ monocytes of study participants with SSc was associated with higher mtDNA copy number in CD14^+^ monocytes, *r* = 0.569, *p* = 0.006*.

**TABLE 2:** The secretion of IL-6 by cultured monocytes of study participants.

IL-6 secretion	Control group	SSc group	Difference, p
Basal secretion, pg/mL	274 (13)	312 (60)	0.008*
LPS-stimulated secretion, pg/mL	33339 (8164)	49652 (14352)	<0.001*

Data presented as Mean (SD).

The mitochondrial activity was analyzed using MitoTracker Orange staining. The mean fluorescence intensity of CD14^+^ monocytes when stained with MitoTracker Orange was 681 (153) in SSc group vs. 685 (89) in control, *p* = 0.897.

## 4 Discussion

The present study quantified changes in mtDNA copy number in patients with SSc compared with apparently healthy individuals without ARD. The results demonstrate a significant increase in mtDNA copy number in CD14^+^ monocytes in patients with SSc compared to controls. Based on our search of the PubMed database, we failed to find published data on the measurements of mtDNA copy number in monocytes from patients with SSc as well as other ARDs. At the same time, insignificant decrease of mtDNA copy number was demonstrated in whole blood of SSc patients. It is known that most studies on mtDNA copy number in diseases associated with mitochondrial dysfunction demonstrate a decrease in mtDNA copy number compared to healthy controls (Ding et al., 2023; Malik, 2023). The previous study on the number of mtDNA copies in the peripheral blood of patients with SSc showed that mtDNA copy number was lower in SSc patients in comparison with healthy participants of the study (Movassaghi et al., 2020). It can be assumed that the differences in mtDNA copy number between SSc and control groups in the current study did not reach statistical significance due to insufficient sample size, while the published study included 46 patients with SSc vs. 49 healthy individuals. However, studies of mtDNA copy number in other ARDs have demonstrated controversial results. In particular, the number of plasma mtDNA copies was increased by 8.8 times in systemic lupus erythematosus compared to the control group (Giaglis et al., 2021). Another recent study demonstrated that mtDNA copy number was statistically higher in blood samples of patients with ankylosing spondylitis in comparison with control subjects (Zhang et al., 2023).

Another important finding of the study was the association of SSc duration and mtDNA copy number in CD14^+^ monocytes. It has been shown that patients with longer duration of SSc have lower mtDNA copy number. The number of mtDNA copies reflects the number of mitochondria in a cell. Presumably, the increase in the number of mitochondria in CD14^+^ monocytes may be a consequence of a compensatory mechanism that is necessary to maintain the reduced function of defective mitochondria in monocytes of patients with shorter duration of SSc. At the same time, in patients with a longer duration of the disease mtDNA copy number decreases due to elimination of dysfunctional mitochondria. In this study mitochondrial activity was analyzed using MitoTracker Orange staining. MitoTracker Orange is a mitochondrial dye which is sensitive to mitochondrial potential that can be used as an indicator of mitochondrial activity and oxidative stress (Tomkova et al., 2018). The difference of fluorescence intensity after MitoTracker Orange staining of CD14^+^ monocytes was not observed between SSc and control despite of increase of mtDNA copy number in monocytes of SSc patients that may explain the compensation of mitochondria function by increasing their number especially taking the fact that mtDNA copy number decreased with disease duration. Mitophagy is a key mechanism aimed to control and remove damaged mitochondria from the cell (Fu et al., 2023). It was previously demonstrated in animal model of SSc using Caveolin-1 (−/−) null mice that mitophagy was increased in the stromal cells of the dermis along with other signs characterizing changes in the skin in SSc (Castello-Cros et al., 2011). The study in patients with another autoimmune disease, Sjögren’s syndrome have shown the decrease of mtDNA copy number and the increase of the expression of genes associated with mitochondrial dynamics in the peripheral blood of patients with Sjögren’s syndrome in comparison with healthy subjects (De Benedittis et al., 2022).

Damaged mitochondria can cause cell death, after which the release of mtDNA contributes to increased systemic inflammation due to the activation of immune cells (Faas and de Vos, 2020). The fact that in this study mtDNA copy number disturbance was demonstrated only in CD14^+^ monocytes, while in whole blood there was no difference in mtDNA copy number between SSc and controls, allows considering mtDNA copy number in CD14^+^ monocytes as a potential indicator of mitochondrial dysfunction of monocytes in SSc and confirms the important role of CD14^+^ monocytes in pathogenesis of SSc. Monocytes have been shown to be important participants in inflammatory and fibrotic processes in SSc through the overproduction of inflammatory cytokines (IL-6, IL-8), chemokines (CCL2, CXCL10), and growth factors (Carvalheiro et al., 2020; Rudnik et al., 2021). The results of the current study demonstrated significantly increased secretion of pro-inflammatory cytokine IL-6 by cultured monocytes of SSc patients under basal and pro-inflammatory conditions in comparison with healthy participants of the study. The increased LPS-stimulated secretion of IL-6 correlated with higher number of mtDNA copies in CD14^+^ monocytes that suggests the important role of mitochondrial dysfunction in pro-inflammatory monocyte activation in SSc pathogenesis. Several recent studies on mtDNA copy number, mitochondrial heteroplasmy and mitophagy in other ARD report that mitochondrial dysfunction is involved in the activation of innate immune system in the pathogenesis of systemic lupus erythematosus and rheumatoid arthritis (Veale et al., 2017; Quintero-González et al., 2022). The recent study on dermal fibroblasts isolated from SSc and healthy donors demonstrated higher levels of mitochondrial reactive oxygen species (ROS) in SSc fibroblasts along with increased secretion of inflammatory cytokines IL-6 and IL-8 induced by oxidative stress (Kizilay Mancini et al., 2022).

The current study had some limitations. The sample size was limited due to low prevalence of SSc, taking that study participants with SSc must not receive glucocorticoid therapy according to the inclusion criteria. This study was a pilot project aimed to assess the phenomenon of mtDNA copy number variation in SSc both in whole blood and CD14^+^ monocytes, so another important limitation of the study was the fact that the investigation of mitochondrial dysfunction parameters such as ROS production, and mitophagy, as well as mitochondrial heteroplasmy were not performed.

The cause of mitochondrial dysfunction may be mitochondrial heteroplasmy. It has been previously shown that mtDNA copy number decreases with age, while the level of mitochondrial heteroplasmy increases (Stifano and Christmann, 2016). The study on the association of mitochondrial heteroplasmy with the number of mtDNA copies, which reflects mitochondrial dysfunction, is a promising direction for further research of the pathogenesis of SSc as well as other ARDs.

## Data Availability

The original contributions presented in the study are included in the article/supplementary material, further inquiries can be directed to the corresponding author.
